# The functional divergence between SPA1 and SPA2 in Arabidopsis photomorphogenesis maps primarily to the respective N-terminal kinase-like domain

**DOI:** 10.1186/s12870-016-0854-9

**Published:** 2016-07-22

**Authors:** Song Chen, Lennart Wirthmueller, Johannes Stauber, Niels Lory, Xu Holtkotte, Lisa Leson, Christian Schenkel, Margaret Ahmad, Ute Hoecker

**Affiliations:** Botanical Institute and Cluster of Excellence on Plant Sciences (CEPLAS), Biocenter, University of Cologne, Zülpicher Str. 47b, 50674 Cologne, Germany; UMR 8256 (B2A) CNRS - UPMC, IBPS, Université Pierre et Marie Curie, Bat C, 9 quai Saint-Bernard, 75252 Paris Cedex 05, France; Present Address: Department of Botany and Plant Biology, University of Geneva, Sciences III, 30 Quai E. Ansermet, 1211 Geneva 4, Switzerland; Present Address: Department of Plant Biochemistry, Dahlem Center of Plant Sciences, Freie Universität Berlin, Königin-Luise-Str. 12-16, Berlin, Germany

**Keywords:** Photomorphogenesis, SPA1, SPA2, COP1, E3 ubiquitin ligase, Functional divergence, Arabidopsis

## Abstract

**Background:**

Plants have evolved complex mechanisms to adapt growth and development to the light environment. The COP1/SPA complex is a key repressor of photomorphogenesis in dark-grown Arabidopsis plants and acts as an E3 ubiquitin ligase to ubiquitinate transcription factors involved in the light response. In the light, COP1/SPA activity is inhibited by photoreceptors, thereby allowing accumulation of these transcription factors and a subsequent light response. Previous results have shown that the four members of the SPA family exhibit partially divergent functions. In particular, SPA1 and SPA2 strongly differ in their responsiveness to light, while they have indistinguishable activities in darkness. The much higher light-responsiveness of SPA2 is partially explained by the much stronger light-induced degradation of SPA2 when compared to SPA1. Here, we have conducted SPA1/SPA2 domain swap experiments to identify the protein domain(s) responsible for the functional divergence between SPA1 and SPA2.

**Results:**

We have individually swapped the three domains between SPA1 and SPA2 - the N-terminal kinase-like domain, the coiled-coil domain and the WD-repeat domain - and expressed them in *spa* mutant Arabidopsis plants. The phenotypes of transgenic seedlings show that the respective N-terminal kinase-like domain is primarily responsible for the respective light-responsiveness of SPA1 and SPA2. Furthermore, the most divergent part of the N-terminal domain was sufficient to confer a SPA1- or SPA2-like activity to the respective SPA protein. The stronger light-induced degradation of SPA2 when compared to SPA1 was also primarily conferred by the SPA2 N-terminal domain. At last, the different affinities of SPA1 and SPA2 for cryptochrome 2 are defined by the N-terminal domain of the respective SPA protein. In contrast, both SPA1 and SPA2 similarly interacted with COP1 in light-grown seedlings.

**Conclusions:**

Our results show that the distinct activities and protein stabilities of SPA1 and SPA2 in light-grown seedlings are primarily encoded by their N-terminal kinase-like domains. Similarly, the different affinities of SPA1 and SPA2 for cry2 are explained by their respective N-terminal domain. Hence, after a duplication event during evolution, the N-terminal domains of SPA1 and SPA2 underwent subfunctionalization, possibly to allow optimal adaptation of growth and development to a changing light environment.

**Electronic supplementary material:**

The online version of this article (doi:10.1186/s12870-016-0854-9) contains supplementary material, which is available to authorized users.

## Background

Plants have evolved complex strategies to adapt to their changing environments. Light is among the most important environmental factors because it serves as the primary source of energy for photosynthesis. Light is also a signaling cue that controls many aspects of plant growth and development, including seed germination, seedling de-etiolation, phototropism, shade avoidance, anthocyanin production and the induction of flowering [1]. Several classes of photoreceptors evolved that constantly monitor light conditions and allow plants to rapidly respond to changing light conditions. These photoreceptors include the red- (R)/far-red- (FR) perceiving phytochromes, blue light (B)-sensing cryptochromes, phototropins and the ZEITLUPE family and the UV-B receptor UVR8 [2–4].

Arabidopsis seedlings grown in the dark display elongated hypocotyls, closed cotyledons and an apical hook. These etiolation phenotypes require the CONSTITUTIVELY PHOTOMORPHOGENIC1/SUPPRESSOR OF PHYA-105 (COP1/SPA) complex. Hence, dark-grown *cop1* mutants and *spa1 spa2 spa3 spa4* quadruple mutants exhibit constitutive de-etiolation, showing the phenotype of light-grown seedlings in darkness [5–7]. The COP1/SPA complex acts as a repressor of photomorphogenesis in the dark, while its repressor function is inhibited by light through multiple mechanisms. B-activated cryptochrome 1 (cry1) interacts with members of the SPA family to disrupt the interaction between COP1 and SPA proteins, leading to reduced COP1/SPA function and de-etiolation of the plant [8, 9]. Similarly, phytochromes interrupt the COP1/SPA interaction in red light [10, 11]. For cry2, COP1/SPA function is reduced by the enhancement of the cry2-COP1 interaction due to cry2-SPA interaction [12]. Another mechanism that inactivates COP1/SPA is based on the light-induced translocation of COP1 from the nucleus to the cytosol [13, 14]. A third mechanism involves the light-induced degradation of SPA1 and SPA2 [15, 16].

The COP1/SPA complex is part of the CULLIN4 (CUL4)-based multi-subunit E3 ubiquitin ligase CUL4-DDB1^COP1/SPA^ [17]. The substrates of this E3 ligase include several transcription factors such as ELONGATED HYPOCOTYL 5 (HY5), LONG HYPOCOTYL IN FR 1 (HFR1) and PRODUCTION OF ANTHOCYANIN PIGMENT (PAP) proteins which are responsible for light-induced photomorphogenesis [18–22]. In the dark, these transcription factors are ubiquitinated by the COP1/SPA-based E3 ubiquitin ligase, leading to their degradation in the 26S proteasome. When the COP1/SPA complex is inhibited by photoreceptors in the light, these transcription factors are stabilized to facilitate their functions in activating the light responses.

COP1 contains an N-terminal RING-finger domain, a coiled-coil domain and C-terminal WD-repeats. The four SPA proteins (SPA1-SPA4) are structurally related to COP1 in that they contain a coiled-coil domain and WD-repeats. However, the N-termini of COP1 and SPAs are distinct, with COP1 having a RING-finger domain and SPAs harboring a kinase-like domain [22, 23]. COP1 and SPAs can form homo- and heterodimers via their respective coiled-coil domains and the COP1/SPA complex forms a tetramer of two COP1 and two SPA proteins. Both COP1 and SPA proteins can interact with most substrates and with DDB1 through their C-terminal WD-repeat domains [17, 24–26]. The function of the SPA kinase-like domain, in contrast, is not well understood. It displays weak sequence similarity to Ser/Thr protein kinases, though many normally invariant amino acids are not conserved in the kinase-like domain of SPA proteins, suggesting that SPA proteins are pseudokinases [27, 28]. Deletion of the large N-terminus of SPA1 including the kinase-like domain retained SPA1 activity in light-grown transgenic *spa1* mutant seedlings, suggesting that the N-terminus of SPA1 is dispensable for SPA1 function [29, 30]. However, functional redundancy with SPA2, SPA3 and SPA4 might mask a need for the N-terminal domain. Indeed, the N-terminus of SPA1 was necessary for full SPA1 activity in flowering time regulation [12]. The N-terminal domain of SPA1 also de-stabilizes SPA1 in light-grown seedlings [29, 30]. Protein-protein interaction studies have demonstrated that the N-terminal domain of SPA1 is essential for binding cry2 in blue light and for binding phytochromes in red light [10, 12], though other domains of SPA1 might also be involved in binding phytochromes [11].

Analysis of *spa* single, double and triple mutants showed that the four *SPA* genes have overlapping but also distinct functions in plant development. In particular, *SPA1* and *SPA2* act redundantly to repress photomorphogenesis in seedlings in the dark, whereas in the light, only *SPA1* serves as a repressor to prevent overstimulation by light. *SPA2*, in contrast, is extremely effectively inactivated by light of even very low fluence rates. As a consequence, *SPA2* has little activity in light-grown plants when compared to *SPA1*, *SPA3* and *SPA4* [5, 23, 31–33]. The molecular basis of the difference between *SPA1* and *SPA2* activity in light-grown seedlings was studied previously. Chimeric *SPA1/SPA2* promoter/cDNA swap experiments have shown that the distinct functions of *SPA1* and *SPA2* genes in light-grown plants are due to differences in the respective protein sequences and independent of the *SPA* promoter sequences. Moreover, we found that the SPA2 protein is more strongly degraded in the light than SPA1 which correlates with the much stronger light-induced repression of SPA2 activity when compared to the activity of SPA1 [15]. Here, we aimed to define the domain(s) in the SPA proteins which are responsible for the distinct activities and stabilities of SPA1 and SPA2 in light-grown seedlings. To this end, we expressed chimeric SPA proteins containing domain swaps of SPA1 and SPA2 in transgenic *spa* mutant plants.

## Results

### Sequence divergence between the N-terminal domains of SPA1 and SPA2 is responsible and sufficient for the functional divergence between SPA1 and SPA2 in the light

**Fig. 1:**
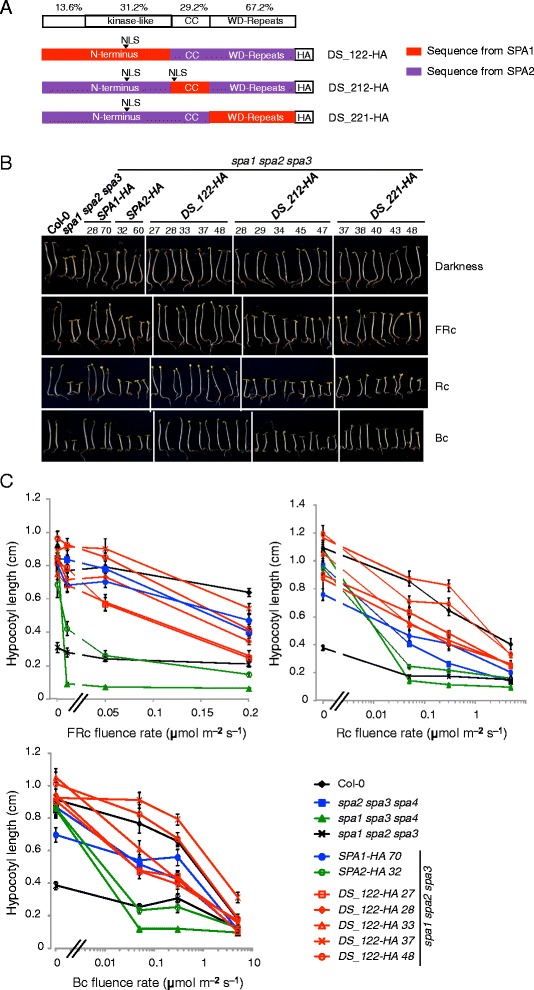
The N-terminal domain is involved in the functional divergence between SPA1 and SPA2. **a** Schematic representation of the chimeric SPA1/SPA2 domain-swap proteins DS_122-HA, DS_212-HA and DS_221-HA. “CC” represents the coiled-coil domain. All chimeric proteins were expressed in a *spa1 spa2 spa3* mutant under the control of the *SPA2* promoter and fused with a C-terminal HA tag. Numbers above the domains indicate the percent identical amino acids between SPA1 and SPA2. NLS indicates the site of a predicted nuclear localization sequence. **b** Phenotype of 4-day-old *spa1 spa2 spa3* mutant *s*eedlings carrying the indicated domain-swap constructs. Representative T2 seedlings are shown. Seedlings expressing *SPA1-HA* or *SPA2-HA* served as controls. All proteins were expressed under the control of the *SPA2* promoter. Seedlings were grown in darkness, 0.05 μmol m^−2^ s^−1^ FRc (FRc), 0.01 μmol m^−2^ s^−1^ Rc (Rc) or 0.05 μmol m^−2^ s^−1^ Bc (Bc) for 4 days. Numbers indicate independent transgenic lines. **c** Quantification of hypocotyl length of 4-day-old DS_122-HA expressing *spa1 spa2 spa3* T3 homozygous seedlings grown under various fluence rates of FRc, Rc or Bc. Error bars represent the SEM of at least 20 seedlings

We previously found that the diverged function of *SPA1* and *SPA2* genes in the light depends on differences in their protein-coding sequences [15]. In order to identify the domain(s) responsible for the SPA1- and SPA2-specific activities of these SPA proteins, we designed three domain swap constructs that encode chimeric SPA proteins with an N-terminal domain, a coiled-coil domain or a WD-repeat domain from SPA1 fused to the remaining domains of SPA2. The chimeric proteins thus contain one domain from SPA1 and two domains from SPA2 (Fig. 1a). They were expressed under the control of the *SPA2* promoter which is constitutively expressed and thus eliminates any transcriptional effects of light on protein function [15, 29]. The chimeric constructs were transformed into the *spa1 spa2 spa3* mutant which exhibits constitutive photomorphogenesis in darkness.

Dark-grown seedlings expressing any of the three chimeric SPA1/SPA2 proteins fully etiolated and thus exhibited full complementation of the *spa1 spa2 spa3* mutant phenotype (Fig. 1b). This was expected because SPA1 and SPA2 do not differ in their functions in darkness [15, 29]. Moreover, this shows that all three chimeric proteins are fully functional and thus not impaired by the domain swap. By contrast, transgenic light-grown seedlings revealed striking differences in phenotype. Expression of the DS_122-HA chimeric protein harboring the N-terminal domain from SPA1 complemented the phenotype of the *spa1 spa2 spa3* mutant to a similar extent as lines expressing SPA1-HA under low fluence rate of FRc, Rc and Bc (Fig. 1b). Hence, swapping the N-terminal domain was sufficient to confer SPA1-like activity to the SPA2 protein. Transgenic seedlings expressing DS_212-HA or DS_221-HA strongly deetiolated under weak FRc, Rc and Bc and thus behaved similarly as the SPA2-HA protein (Fig. 1b). These results indicate that the coiled-coil domain and the WD-repeat domain of SPA1 are not sufficient to confer a SPA1-like activity to an otherwise SPA2 protein. In summary, the distinct functions of SPA1 and SPA2 in light-grown seedlings can be mapped to the respective N-terminal domain, while the coiled-coil domains and the WD-repeat domains are functionally similar and interchangeable between SPA1 and SPA2.

Based on these findings, we generated homozygous transgenic lines expressing the chimeric DS_122-HA protein and further characterized their responses to Rc, FRc and Bc of different fluence rates. With increasing fluence rate of FRc, Rc and Bc, seedlings of *DS_122-HA*-expressing *spa1 spa2 spa3* lines exhibited only a moderate reduction in hypocotyls length and thus behaved similarly to SPA1-HA-expressing seedlings (Fig. 1c). In contrast, seedlings expressing SPA2-HA responded to light with an extreme shortening of their hypocotyls. These transgenic seedlings deetiolated to a similar extent as the *spa1 spa2 spa3* progenitor, indicating that there was no residual SPA2 activity present in the light, as was reported previously [15]. These results confirm that the SPA1 N-terminal domain is sufficient to maintain repressor activity in an otherwise SPA2 protein in light-grown seedlings.

### The divergent region of the N-terminal domain is responsible and sufficient for the functional divergence between SPA1 and SPA2 in the light

**Fig. 2:**
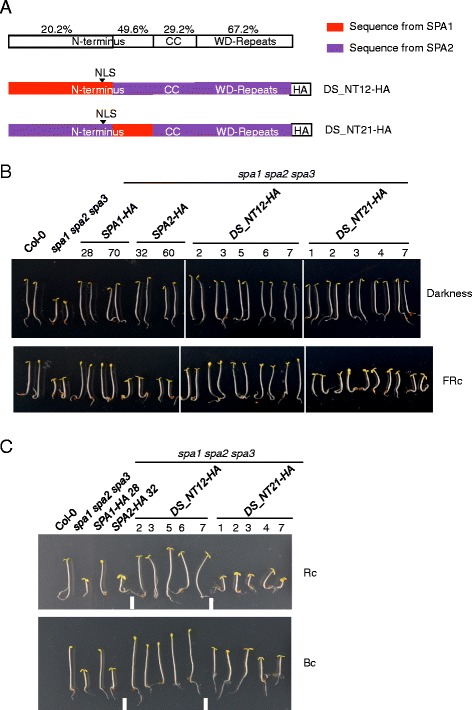
The diverged part of the N-terminal domain of SPA contributes to the divergence of SPA1/SPA2 function in the light. **a** The top row shows a schematic representation of amino acid identity between SPA1 and SPA2 domains. Below indicates the chimeric SPA1/SPA2 domain swap proteins DS_NT12-HA and DS_NT21-HA carrying swaps in the N-terminal domains of SPA1/SPA2. All chimeric constructs were expressed in a *spa1 spa2 spa3* mutant background under the control of the *SPA2* promoter and fused with a C-terminal HA tag. NLS indicates the site of a predicted nuclear localization sequence. **b**, **c** Phenotype of 4-day-old *spa1 spa2 spa3* mutant seedlings expressing *DS_NT12-HA* or *DS_NT21-HA* swap constructs. Representative T2 seedlings are shown. Seedlings were grown in darkness or in 0.05 μmol m^−2^ s^−1^ FRc (**b**) and in 0.01 μmol m^−2^ s^−1^ Rc or 0.05 μmol m^−2^ s^−1^ Bc (**c**) for 4 days. Numbers indicate independent transgenic lines

We subsequently aimed to narrow down the region in the N-terminal domain of SPA1 and SPA2 that is responsible for the divergence of SPA1 and SPA2 function in the light. Based on sequence similarity, the N-termini of SPA1 and SPA2 were divided into two parts and swapped within the SPA2 protein (Fig. 2a). The N-terminal part is very divergent between SPA1 and SPA2, whereas the sequence of the C-terminal part is quite conserved among all four SPA proteins from Arabidopsis and also with SPAs from other species including Physcomitrella. The latter also shows moderate sequence similarity with Ser/Thr protein kinases [27, 28, 34].

Dark-grown *spa1 spa2 spa3* mutant seedlings expressing chimeric DS_NT12 or DS_NT21 proteins displayed a fully etiolated phenotypes, indicating that the chimeric proteins complemented the *spa1 spa2 spa3* mutant phenotype and thus were fully functional similar to the non-chimeric SPA1 and SPA2 proteins (Fig. 2b). In FRc, the transgenic seedlings displayed distinct phenotypes: the hypocotyl lengths of the *DS_NT12-HA* lines were similar to those of SPA1-HA expressing lines, while the hypocotyl lengths of *DS_NT21-HA* lines were similar to those of SPA2-HA expressing lines (Fig. 2b). Similar results were obtained in Rc and Bc (Fig. 2c). These findings indicate that swapping the more diverged part of the N-terminal domain was sufficient to confer a SPA1-like activity to the SPA2 protein.

### The WD-repeat domain can also contribute to the functional divergence between SPA1 and SPA2 in the light

**Fig. 3:**
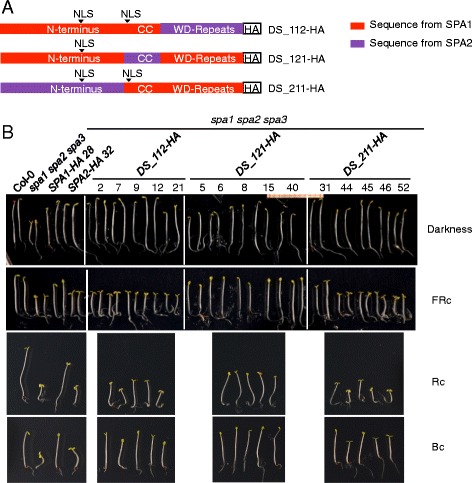
WD-repeat domain is involved in the functional divergence of SPA1 and SPA2. **a** Schematic representation of chimeric SPA1/SPA2 domain swap *DS_112-HA*, *DS_121-HA* and *DS_211-HA* constructs. All chimeric constructs were expressed in a *spa1 spa2 spa3* mutant background under the control of the *SPA2* promoter and fused with a C-terminal HA tag. NLS indicates the location of a predicted nuclear localization sequence. **b** Phenotype of 4-day-old *spa1 spa2 spa3* seedlings expressing *DS_112-HA*, *DS_121-HA* and *DS_211-HA* domain swap constructs. Representative T2 seedlings are shown. Seedlings were grown in darkness or in 0.05 μmol m^−2^ s^−1^ FRc, 0.01 μmol m^−2^ s^−1^ Rc or 0.05 μmol m^−2^ s^−1^ Bc for 4 days. Numbers indicate independent transgenic lines

After examining the domain swaps containing one domain from SPA1 and two domains from SPA2, we conducted the reverse experiment by introducing one domain from SPA2 into an otherwise SPA1 protein (Fig. 3a). These chimeric proteins were also expressed under the control of the *SPA2* promoter in a *spa1 spa2 spa3* mutant. All three constructs - *DS_112-HA*, *DS_121-HA* and *DS_211-HA* - complemented the seedling phenotype of the *spa1 spa2 spa3* mutant in the dark, again indicating that the domains from SPA1 and SPA2 are compatible with each other and that the chimeric proteins are fully functional in darkness (Fig. 3b). In the light, the phenotypes of the transgenic seedlings differed. FRc-grown seedlings expressing DS_112-HA or DS_211-HA exhibited short hypocotyls and open cotyledons similar to the *spa1 spa2 spa3* progenitor and SPA2-HA-expressing *spa1 spa2 spa3* seedlings, indicating that these chimeric proteins, like SPA2, retain barely any activity in the light. Similar results were obtained in Rc and - to a lesser extent, in Bc (Fig. 3b). In contrast, seedlings expressing DS_121-HA showed strong etiolation and behaved similar to the SPA1-HA protein (Fig. 3b). Hence, swapping the coiled-coil domain from SPA2 into the SPA1 protein did not alter SPA1 activity. A similar observation was made previously when introducing the coiled-coil domain from SPA2 into SPA1 [30]. Hence, the coiled-coil domains from SPA1 and SPA2 appear equivalent.

Taken together, these results indicate that both the N-terminal domain and the WD-repeat domain of SPA2 can confer SPA2-like function to an otherwise SPA1 protein. Hence, both the N-terminal domain and the WD-repeat domain, but not the coiled-coil domain, can play a role in the distinct functions of SPA1 and SPA2.

### The N-terminal domains of SPA1 and SPA2 differentially regulate SPA protein stability

**Fig. 4:**
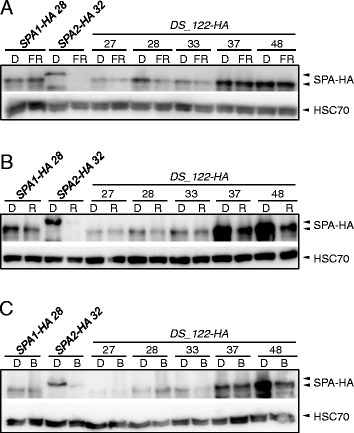
The SPA1 N-terminal domain provides the higher stability to the chimeric DS_122-HA protein in the light. **a**-**c** SPA-HA protein levels in 4-day-old T3 homozygous transgenic *spa1 spa2 spa3* mutant seedlings grown in darkness (D) and subsequently transferred to 0.35 μmol m^−2^ s^−1^ FR (**a**), 0.01 μmol m^−2^ s^−1^ R (**b**) or 0.1 μmol m^−2^ s^−1^ B (**c**) for 30 min. SPA-HA was detected using an α–HA antibody. HSC70 levels served as a loading control. Numbers indicate independent transgenic lines

Our previous studies showed that the SPA2 protein is more rapidly degraded in the light when compared to the SPA1 protein [15]. We therefore used domain swap lines to test which domains contribute to the differential protein stability of SPA1 and SPA2. DS_122-HA protein levels changed only moderately upon irradiation of dark-grown seedlings with FRc, Rc or Bc (Fig. 4). Similarly, the SPA1-HA protein was relatively stable after this short exposure to light, while the SPA2-HA protein was fully degraded upon light exposure, as reported previously [16]. Hence, the DS_122-HA protein behaved similarly to the SPA1 protein, indicating that the N-terminal domain of SPA1 stabilizes the chimeric DS_122-HA protein in the light.

Levels of the DS_212-HA protein harboring the coiled-coil domain of SPA1 in a SPA2 background strongly decreased in response to light (Additional file 1: Figure S1A). This demonstrates that the coiled-coil domains of SPA1 and SPA2 are functionally equivalent with respect to protein stability. The levels of DS_221-HA harboring the WD-repeat domain of SPA1 in a SPA2 background strongly decreased in response to light, suggesting that the WD-repeats of SPA1 and SPA2 also do not severely differ in their effects on protein stability in this configuration (Additional file 1: Figure S1B). In summary, only a swap-in of the SPA1 N-terminal domain can confer higher stability to a SPA2 protein in the light.

When conducting the reverse experiment, i.e. swapping a SPA2 domain into a SPA1 protein, the N-terminal domain of SPA2 de-stabilized the SPA1 protein in light-grown DS_211-HA lines when compared to SPA1-HA lines (Additional file 2: Figure S2A). Swapping the coiled-coil domain did not dramatically alter protein stability in two out of three lines (Additional file 2: Figure S2B), while swapping the WD-repeat domain tended to destabilize the protein (Additional file 2: Figure S2C). Hence, in an otherwise SPA1 protein, introducing the N-terminal or the WD-repeat domain of SPA2 strongly or moderately destabilized the chimeric protein, respectively.

### SPA1 and SPA2 have similar affinities for cry1 in blue light

**Fig. 5:**
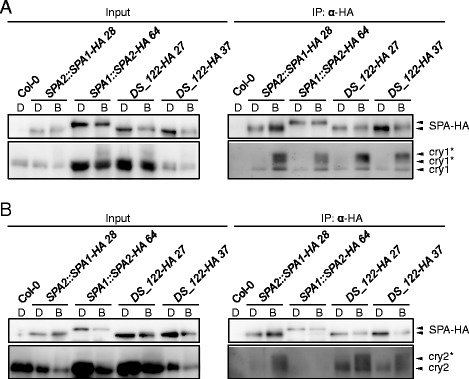
DS_122-HA associates with cry1 and cry2 in blue light. **a**, **b** Co-immunoprecipitation of cry1 (**a**) and cry2 (**b**) by DS_122-HA. 4-day-old dark-grown seedlings (D) were transferred to 50 μmol m^−2^ s^−1^ B for 1 h (B). DS-122-HA was expressed in *spa1 spa2 spa3* and under the control of the *SPA2* promoter. Col-0, *SPA2::SPA1-HA* and *SPA1::SPA2-HA* lines were used as controls. SPA-HA proteins were immunoprecipitated using α-HA beads. α-HA antibody was used to detect SPA-HA proteins. α-cry1 and α-cry2 antibodies were used to detect cry1 and cry2, respectively. Asterisks likely indicate phosphorylated cry1 and cry2. To obtain similar SPA-HA protein levels from different transgenic lines in B, all seedlings were treated with proteasome inhibitor to reduce protein degradation in B. Also, SPA2-HA was expressed under the control of the stronger *SPA1* promoter to counteract the strong B-induced degradation of SPA2. Five times more protein extract was used for the *SPA1::SPA2-HA 64* and *DS_122-HA 27* co-immunoprecipitations than for th*e SPA2::SPA1-HA 28* and *DS_122-HA 37* co-immunoprecipitations

Since SPA2 is much more strongly inactivated by blue light than SPA1 [15, 29] we tested whether SPA2 might interact with cry1 more strongly than SPA1. Figure 5a shows that SPA1 and SPA2 have similar affinities for cry1 in vivo. This suggest that the higher responsiveness of *spa1 spa3 spa4* mutants to blue light when compared to *spa2 spa3 spa4* mutants [29] is not due to a differential cry1 binding strength. This finding is consistent with our previous observation that inactivation of SPA2 is primarily mediated by phyA [16].

### The N-termini of SPA1 and SPA2 differentially interact with cry2

We previously showed that SPA2 does not bind cry2 in vivo, while SPA1 does interact with cry2 in vivo, as was shown previously [12, 16]. We therefore asked whether swapping of the SPA1 N-terminal domain into SPA2 blocked the interaction between the chimeric SPA protein and cry2. Figure 5b shows that the DS_122-HA chimeric protein was able to associate with cry2 in B, while SPA2-HA was not. Hence, the differential affinity of SPA1 and SPA2 for cry2 is due to the distinct sequences in their N-terminal domains.

### SPA1 and SPA2 similarly interact with COP1 in light-grown seedlings

**Fig. 6:**
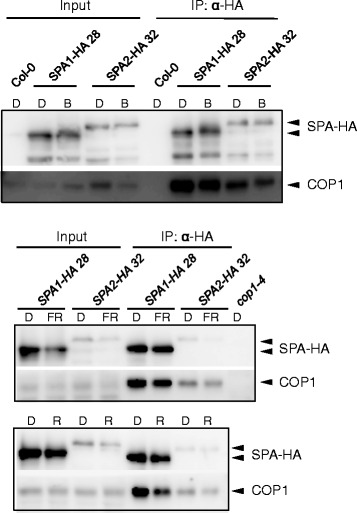
SPA1 and SPA2 similarly interact with COP1 in dark-grown vs. light-grown seedlings. Co-immunoprecipitation of COP1 by SPA1-HA and SPA2-HA in the 4-day-old *spa1 spa2 spa3* seedlings expressing SPA1-HA and SPA2-HA proteins under the control of the native SPA2 promoter. Seedlings were grown in darkness and were subsequently transferred to 0.05 μmol m^−2^ s^−1^ FR or R for 2 h or 50 μmol m^−2^ s^−1^ B for 1 h. Proteasome inhibitor was added to prevent degradation of SPA proteins in blue light. SPA1-HA and SPA2-HA proteins were immunoprecipitated using α-HA beads. Col-0 was used as a negative control. α-HA antibody was used to detect SPA-HA proteins. α-COP1 antibody was used to detected COP1. Equal amount of total protein was used as inputs

We subsequently asked whether light is more effective in disrupting a COP1/SPA2 complex when compared to a COP1/SPA1 complex. SPA1 and SPA2 co-immunoprecipitated the same amount of COP1 in dark- and light-grown seedlings (Fig. 6). Hence, no differential effect of light on COP1/SPA1 and COP1/SPA2 complex abundance was observed.

## Discussion

The COP1/SPA protein complex is an important negative regulator of photomorphogenesis in dark-grown Arabidopsis plants. Our previous analysis of *SPA* genes revealed that the four *SPAs* have at least partially distinct functions during plant growth and development. In particular, SPA1 and SPA2 proteins strongly differ in their responsiveness to light, while they have indistinguishable activities in darkness. In light-grown seedlings, SPA1 retains considerable repressor activity to prevent overstimulation by light, while SPA2 is almost fully inactivated by even very low fluences of light [5, 15, 29]. Here, we have shown that the stronger light-responsiveness of SPA2 when compared to SPA1 maps primarily to the N-terminal domain which includes the kinase-like domain. This conclusion is based on our finding that swapping the N-terminal domain of SPA1 into a SPA2 protein confers SPA1-like responsiveness to R, FR and B in the chimeric protein. The same result was found when conducting the reverse experiment, i.e. when introducing the N-terminus of SPA2 into an otherwise SPA1 protein. This finding is consistent with the observation that sequence divergence between SPA1 and SPA2 is highest in the N-terminal domain (73 %) when compared to the WD-repeat domain (33 %) and the coiled-coil domain (71 %). When we further divided the N-terminal domain into a smaller, highly conserved part and a larger, not conserved part, we found that the non-conserved part of SPA1 and SPA2 conferred the SPA1- or SPA2-specific light-responsiveness, respectively. Hence, these specific SPA activities that map to the N-terminal domain evolved through major sequence alterations and not through smaller sequence changes in a highly conserved domain. It remains to be established which of the two *SPA* genes, *SPA1* or *SPA2*, evolved a new activity since the duplication event leading to *SPA1* and *SPA2* [35]. Since *SPA3* and *SPA4* respond to light similarly to *SPA1*, it is likely that the extreme light-induced inhibition of SPA2 is the recent evolutionary innovation.

Though the N-terminal domain is the primary domain responsible for the distinct activities of SPA1 and SPA2 in light-grown seedlings, the WD-repeat domain can also contribute at least to some extent to the functional divergence between SPA1 and SPA2. We found that swapping the WD-repeat domain from SPA2 into a SPA1 protein also strongly enhanced light-responsiveness of the chimeric protein. However, the reverse experiment, i.e. introducing the WD-repeat from SPA1 into a SPA2 protein did not reduce light-responsiveness. Hence, the SPA-specific effect of the WD-repeat might be context-dependent. However, it is also possible that the enhanced light-induced inactivation of this chimeric DS_112 protein is due to a partial loss-of-function caused by the domain swap per se. This partial loss-of function might only be detectable in light-grown seedlings, while the activity of the chimeric protein might be sufficient for full suppression of photomorphogenesis in darkness. Indeed, such a phenotype was observed in the non-constitutively photomorphogenic *cop1*^*eid6*^ mutant [36]. Moreover, it is known that SPA activities are more limiting in light-grown seedlings than in dark-grown seedlings since *spa* single mutants exhibit a mutant phenotype only in the light [34, 37]. In total, we therefore conclude that the functional divergence between SPA1 and SPA2 primarily maps to the divergent part of the N-terminal domain.

We considered at least two, not mutually exclusive mechanisms that might contribute to the distinct light-responsiveness of SPA1 and SPA2 proteins. First, the N-termini of SPA1 and SPA2 might have distinct affinities for photoreceptors. There is no evidence that differences in the interactions with cryptochromes might be causal for the higher light-responsiveness of SPA2. Our results show that SPA1 and SPA2 interacted similarly with cry1 in B. With respect to cry2, SPA2 has a severely reduced - rather than increased - affinity for cry2 when compared to SPA1 [16]. Similarly, we have shown here that the N-terminal domain swap protein DS_122 conferring a lower responsiveness to light exhibits a restored interaction with cry2 when compared to SPA2. The relative in vivo affinities of SPA1 and SPA2 for phytochromes are thus far unknown. It is known, however, that the N-terminal domain of SPA1 which is responsible for the distinct functions of SPA1 and SPA2 interacts with phyA in the yeast-two hybrid system [10]. Also, phytochromes are the primary photoreceptors inactivating SPA2 in R, FR as well as B [16]. On the other hand, SPA2 interacted less with phyA in R-treated yeast cells when compared with SPA1 [10]. Hence, further experiments are necessary to resolve whether differential interactions of SPA1 and SPA2 with phytochromes contribute to the distinct light-responsiveness of SPA1 and SPA2.

Even if affinities for the photoreceptors themselves do not account for the functional divergence of SPA1 and SPA2, downstream effects in repressing COP1/SPA1 and COP1/SPA2 function by photoreceptors may differ and be causative for the distinct light-responsiveness of SPA1 and SPA2. One mechanism by which light inactivates COP1/SPA1 and COP1/SPA2 function is the phytochrome-induced degradation of SPA1 and SPA2. Indeed, SPA2 is much more effectively degraded in the light than SPA1 [15, 16]. Moreover, the degree of photomorphogenesis in transgenic lines expressing chimeric SPA1/SPA2 proteins was negatively correlated with the stability of the chimeric proteins in the light, i.e. highly responsive lines expressed a chimeric protein that is unstable in the light, and vice versa. This correlation supports the notion that light-induced SPA protein degradation is intrinsically related to the light-induced inhibition of COP1/SPA activity. Our finding that a swap of the SPA1/SPA2 N-terminal domains strongly alters the stability of the chimeric SPA proteins also agrees with previous observations showing that the N-terminal domain of SPA1 de-stabilizes the SPA1 protein [29, 30]. Light inactivates COP1/SPA function also by dissociation of the COP1/SPA interaction and by nuclear exclusion of COP1 [22]. Hence, one can hypothesize that the light-induced dissociation of a COP1/SPA2 complex might be more sensitive to light than the dissociation of a COP1/SPA1 complex, and this difference might depend on the N-terminal sequence in SPA. Our results do not provide evidence for this idea because both SPA1 and SPA2 were in a complex with COP1 also in light-treated seedlings. However, since the light-induced disruption of the COP1/SPA complex is much less detectable in co-immunoprecipitation assays than in yeast three-hybrid or FRET-FLIM studies [8–11], other assays may be necessary to compare the light-induced rearranging in the respective COP1/SPA complexes.

## Conclusions

In summary, our results have defined the N-terminal domains of SPA1 and SPA2 as a "light-sensitivity" domain that confers the distinct light-responsiveness to the respective SPA protein regarding light-induced photomorphogenesis and light-induced SPA degradation. The exact mechanism in which the N-terminal domain is involved and which role photoreceptors play remain to be resolved.

## Methods

### Plant material, light sources and growth conditions

Wild-type *Arabidopsis thaliana* Col-0 and the *spa* triple mutants *spa1-7 spa2-1 spa3-1*, *spa2-1 spa3-1 spa4-1* and *spa1-7 spa3-1 spa4-1* were used [29]. Transgenic lines expressing full-length SPA1 or SPA2 proteins under the control of the *SPA2* promoter were *SPA2::SPA1-HA 28* and *SPA2::SPA1-HA 70* or *SPA2::SPA2-HA 32* and *SPA2::SPA2-HA 60,* respectively, as described previously [15]. LED light sources and growth conditions were as described previously [5].

### Generation of transgenic plants expressing domain swap constructs

The *DS_122-HA* lines express a chimeric protein with amino acids 1–552 from SPA1 and 580–1036 from SPA2. The *DS_212-HA* lines express a chimeric protein with amino acids 1–571 from SPA2, 545–703 from SPA1 and 711–1036 from SPA2. The *DS_221-HA* lines express a chimeric protein with amino acids 1–702 from SPA2 and 696–1029 from SPA1. The *DS_112-HA* lines express a chimeric protein with amino acids 1–703 from SPA1 and 711–1036 from SPA2. The *DS_121-HA* lines express a chimeric protein with amino acids 1–552 from SPA1, 580–702 from SPA2 and 696–1029 from SPA1. The *DS_211-HA* lines express a chimeric protein with amino acids 1–571 from SPA2 and 545–1029 from SPA1. Two highly conserved regions were selected for domain swapping. The first motif lies between N-terminal domain and the coiled-coil domain and reads SELLLHFL for SPA1 and SELLQHFL for SPA2. The second motif lies between the coiled-coil domain and the WD-repeats and reads ARYSKFET which is identical in both SPA1 and SPA2 proteins.

To generate these domain-swap constructs, first of all, a 4 kb Apal Sall fragment comprising the full-length *SPA2* ORF, the C-terminal triple HA tag and the *SPA2* 3´ UTR was sub-cloned into *pBS SK+* resulting the *pBS SK+ cSPA2* construct. Next, in order to generate domain swap constructs expressing one SPA1 domain and two SPA2 domains (DS_122, DS_212, DS_221) sequences encoding N-terminus, coiled-coil or WD-repeats from SPA1 were amplified from *pJHA212-hpt SPA2::SPA1-HA* [15] by PCR using the primer pairs LW4 and LW5, LW6 and LW7 and LW8 and LW9, respectively, which are compatible with the conserved regions used for swapping. The resulting PCR fragments were gel-purified. The mutagenesis PCRs which create the respective domain swap constructs were performed according to the 'Quik Change Mutagenesis Kit' (Agilent Technologies) using combinations of the template plasmid *pBS SK+ cSPA2* and the gel-purified PCR products serving as primers. In order to generate swapping constructs expressing two SPA1 domains and one SPA2 domain, sequences encoding N-terminus, coiled-coil or WD-repeats from SPA1 were PCR-amplified from the plasmid *pJHA212-hpt SPA2::SPA1-HA* [15] using the primer pairs LW4 and SC_ds_5 primers, LW6 and SC_ds_7 and LW8 and LW9, respectively, which are compatible with the conserved regions used for swapping. The resulting PCR fragments were gel-purified. The mutagenesis PCRs were performed using a combination of the template plasmid which already expresses the chimeric proteins containing one SPA1 domain and two SPA2 domains generated previously and the newly gel-purified PCR fragments serving as primers. For generating the construct expressing the N-terminus and coiled-coil from SPA1 and the WD-repeats from SPA2 (DS_112), the purified SPA1 N-terminus PCR product was combined with the domain swap construct generated previously expressing the coiled-coil domain from SPA1 and N-terminal domain and WD-repeats from SPA2. For generating the construct expressing both coiled-coil and WD-repeats from SPA1 and N-terminal domain from SPA2 (DS_211), the purified SPA1 coiled-coil PCR product was combined with the domain swap construct generated previously expressing the WD-repeats of SPA1 and N-terminal domain and coiled-coil of SPA2. For generating the construct expressing both N-terminal domain and WD-repeats of SPA1 and coiled-coil of SPA2 (DS_121), the purified SPA1 WD-repeats PCR product was combined with the domain swap construct generated previously expressing the N-terminal domain of SPA1, coiled-coil and WD-Repeats of SPA2.

To swap the SPA1 amino acids 1–425 with SPA2 amino acids 1–450 or SPA1 amino acids 426–552 with SPA2 amino acids 451–579 in a SPA2 background, generating the *DS_NT12-HA and DS_NT21-HA* lines respectively, motifs between less conserved N-terminal regions and more conserved C-terminal regions in the N-termini of SPA1 and SPA2 were chosen. In SPA1, the selected motif was LSVSSVSRKQSM while in SPA2, the motif was HCSTVACPFTSV. Sequences encoding amino acids 1–425 and 426–552 of the SPA1 N-terminus were amplified from *pJHA212-hpt SPA2::SPA1-HA* [15] by PCR using the primer pairs LW4 and SC-P2 and SC-P3 and LW5, respectively, which are compatible with the motifs selected for swapping. The resulting PCR fragments were gel-purified. The mutagenesis PCRs which generate the swapping constructs were performed using the combination of the template plasmid *pBS SK+ cSPA2* and the gel-purified PCR products serving as primers.

All mutagenesis PCRs were performed using Pfu polymerase to extend and incorporate the mutagenic primers resulting in nicked circular strands. The products were digested with DpnI to remove methylated, non-mutated parental templates. After digestion, the products were transformed into *DH5α* and plated on LB agar plates containing 100 μg/ml ampicillin. Plasmid DNAs were purified from selected colonies and sequenced. Confirmed swapping constructs were cloned back into *pJHA212-hpt SPA2::SPA2-HA* [15] via ApaI and SalI sites to replace the full length *SPA2*. The final constructs were transformed into *spa1-7 spa2-1 spa3-1* mutant plants by floral dipping. T2 or T3 plants were used for analysis. All primers used for cloning are listed in the Additional file 3: Table S1.

### Isolation of total proteins and immunoblot analysis

Total proteins from dark-grown and light-treated transgenic seedlings were isolated and used for immunoblot analysis as described previously [16].

### Co-immunoprecipitations

Co-immunoprecipitations for examining SPA-cry and SPA-COP1 associations in B were performed using μMACS Anti-HA Starting Kits (Miltenyi Biotec) as described previously and according to the manufacturer’s protocol with minor modification [16]. Prior to the light treatment, seedlings were pre-incubated in 100 μM MG132 and 10 μM clasto-lactacystin β-lactone twice, 15 min each, to prevent degradation of SPA proteins. Five times more protein extract was used for the *SPA1::SPA2-HA 64* and *DS_122-HA 27* line than for the *SPA2::SPA1-HA 28* and *DS_122-HA 37* line. Co-immunoprecipitations for examining SPA-COP1 association in FR and R were performed using Anti-HA Affinity Matrix (Roche) according to the manufacturer’s protocol. Equal amount of total protein was used as inputs.

### Hypocotyl length measurement

Flattened seedlings were imaged by a Nikon D5000 digital camera on MS plates. Measurements were conducted using ImageJ 1.43u (Wayne Rasband, National Institutes of Health).

## Abbreviations

B, blue; COP1, CONSTITUTIVELY PHOTOMORPHOGENIC1; cry1, cryptochrome 1; cry2: cryptochrome 2; CUL4, cullin 4; DDB1, damaged DNA binding protein 1; eid6, empfindlicher im dunkelroten Licht 6; FLIM, fluorescence lifetime imaging; FR, far-red; FRET, fluorescence resonance energy transfer; HA, human influenza hemagglutinin; HFR1, long hypocotyl in far-red 1; HY5, long hypocotyl 5; ORF, open reading frame.; PAP, production of anthocyanin pigment; phyA, Phytochrome A; R, red; RING, really interesting new gene*;* SPA, SUPPRESSOR OF PHYA-105; UV-B, ultraviolet B; UVR8, UV resistance locus8

## Additional files

Additional file 1: Figure S1. The coiled-coil and WD-repeat domains of SPA1 do not provide higher stability to the chimeric SPA2 protein in light-grown seedlings. **A**, **B**. SPA-HA protein levels in 4-day-old T2 *DS_212-HA* (**A**) or *DS_221-HA* (**B**) transgenic *spa1 spa2 spa3* mutant seedlings. Seedlings were grown in darkness (D) for 4 days and subsequently transferred to 0.35 μmol m^−2^ s^−1^ FR for 30 min. All transgenes were expressed under the control of the *SPA2* promoter. SPA-HA was detected using an α–HA antibody. HSC70 levels served as a loading control. (PDF 245 kb) Additional file 2: Figure S2. The N-terminal domain and the WD-repeat domain of SPA2 render the chimeric SPA1 proteins more unstable in light-grown seedlings. **A**-**C**. SPA-HA protein levels in 4-day-old T2 *DS_211-HA* (**A**), *DS_121-HA* (**B**) and *DS_112-HA* (**C**) transgenic *spa1 spa2 spa3* seedlings. Seedlings were grown in darkness (D) for 4 days and subsequently transferred to 0.35 μmol m^−2^ s^−1^ FR for 30 min. All transgenes were expressed under the control of the *SPA2* promoter. SPA-HA was detected using an α–HA antibody. HSC70 levels served as a loading control. (PDF 349 kb) Additional file 3: Table S1. Primer sequences. (PDF 32 kb)
