# Supplementary material for: The functional divergence between SPA1 and SPA2 in Arabidopsis photomorphogenesis maps primarily to the respective N-terminal kinase-like domain
Source: BMC Plant Biol. 2016 Jul 22;16:165. doi: 10.1186/s12870-016-0854-9 (PMC4957354; doi:10.1186/s12870-016-0854-9)
Supplement: Additional file 1: Figure S1. — The coiled-coil and WD-repeat domains of SPA1 do not provide higher stability to the chimeric SPA2 protein in light-grown seedlings. A, B. SPA-HA protein levels in 4-day-old T2 DS_212-HA (A) or DS_221-HA (B) transgenic spa1 spa2 spa3 mutant seedlings. Seedlings were grown in darkness (D) for 4 days and subsequently transferred to 0.35 μmol m−2 s−1 FR for 30 min. All transgenes were expressed under the control of the SPA2 promoter. SPA-HA was detected using an α–HA antibody. HSC70 levels served as a loading control. (PDF 245 kb) [file 12870_2016_854_MOESM1_ESM.pdf]

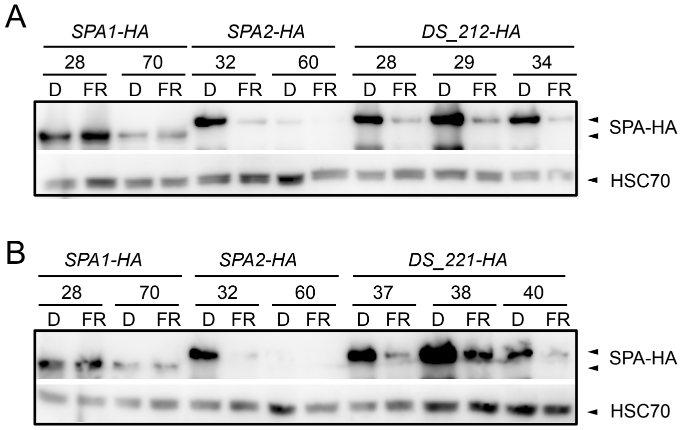

Figure S1. The coiled-coil and WD-repeat domains of SPA1 do not provide higher stability to the chimeric SPA2 protein in light-grown seedlings.

A, **B**. SPA-HA protein levels in 4-day-old T2 *DS_212-HA* (**A**) or *DS_221-HA* (**B**) transgenic *spa1 spa2 spa3* mutant seedlings. Seedlings were grown in darkness (D) for 4 days and subsequently transferred to 0.35 µmol m^{-2} s^{-1} FR for 30 min. All transgenes were expressed under the control of the *SPA2* promoter. SPA-HA was detected using an α–HA antibody. HSC70 levels served as a loading control.
