# Supplementary material for: The functional divergence between SPA1 and SPA2 in Arabidopsis photomorphogenesis maps primarily to the respective N-terminal kinase-like domain
Source: BMC Plant Biol. 2016 Jul 22;16:165. doi: 10.1186/s12870-016-0854-9 (PMC4957354; doi:10.1186/s12870-016-0854-9)
Supplement: Additional file 2: Figure S2. — The N-terminal domain and the WD-repeat domain of SPA2 render the chimeric SPA1 proteins more unstable in light-grown seedlings. A-C. SPA-HA protein levels in 4-day-old T2 DS_211-HA (A), DS_121-HA (B) and DS_112-HA (C) transgenic spa1 spa2 spa3 seedlings. Seedlings were grown in darkness (D) for 4 days and subsequently transferred to 0.35 μmol m−2 s−1 FR for 30 min. All transgenes were expressed under the control of the SPA2 promoter. SPA-HA was detected using an α–HA antibody. HSC70 levels served as a loading control. (PDF 349 kb) [file 12870_2016_854_MOESM2_ESM.pdf]

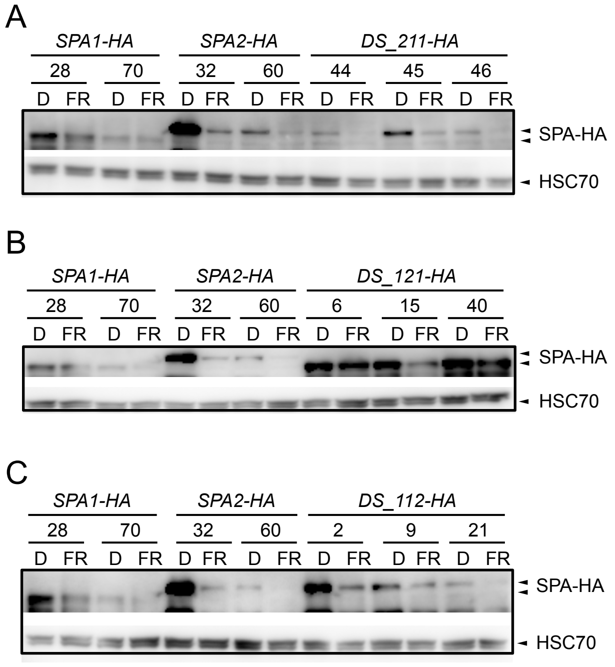


**Figure S2.** The N-terminal domain and the WD-repeat domain of SPA2 render the chimeric SPA1 proteins more unstable in light-grown seedlings.

**A**-**C**. SPA-HA protein levels in 4-day-old T2 *DS_211-HA* (**A**), *DS_121-HA* (**B**) and *DS_112-HA* (**C**) transgenic *spa1 spa2 spa3* seedlings. Seedlings were grown in darkness (D) for 4 days and subsequently transferred to 0.35 µmol $m^{-2}$ $s^{-1}$ FR for 30 min. All transgenes were expressed under the control of the *SPA2* promoter. SPA-HA was detected using an α–HA antibody. HSC70 levels served as a loading control.
