# Supplementary material for: The functional divergence between SPA1 and SPA2 in Arabidopsis photomorphogenesis maps primarily to the respective N-terminal kinase-like domain
Source: BMC Plant Biol. 2016 Jul 22;16:165. doi: 10.1186/s12870-016-0854-9 (PMC4957354; doi:10.1186/s12870-016-0854-9)
Supplement: Additional file 3: Table S1. — Primer sequences. (PDF 32 kb) [file 12870_2016_854_MOESM3_ESM.pdf]

Table S1: Primer sequences

Primer name	Sequence (5´ to 3´)
LW4	ATAGGGCGAATTGGGTACCGGGCCCATGCCTGTTATGGAAAGAG
LW5	TCTGCCTTTTCTCTTGGGACAAGAACAAAAAATGAAGTAACAG
LW6	ATCCATTGAACAAGAGGACACAGAGTCTGAGCTGTTACTTCATT
LW7	ACTCACTGGTCCTCAGCACACCCCGGGTTTCGAACTTGCTATA
LW8	GTTCTTTGATGGGTTATGCAAATATGCTCGGTATAGCAAGTTCG
LW9	GAACGTCATATGGGTAAGCGGCCGCAACAAGTTTTAGTAGCTT
SC_ds_5	TCTTCTTCTGCACTTCTAATGA
SC_ds_7	GGTCCCCACTTCTTATTGTCCC
SC-P2	CAACTGCTCACTCACTGAAGTAAAAGACACGCTTAAATTTTTTATGTA
SC-P3	CATTGTAGTACAGTTGCCTGCCCTTCAGTGTCGAGAAAACAGTCTATG
